# Protective Effects of White Kidney Bean (*Phaseolus vulgaris* L.) against Diet-Induced Hepatic Steatosis in Mice Are Linked to Modification of Gut Microbiota and Its Metabolites

**DOI:** 10.3390/nu15133033

**Published:** 2023-07-04

**Authors:** Qiqian Feng, Zhitao Niu, Siqi Zhang, Li Wang, Lijun Dong, Dianzhi Hou, Sumei Zhou

**Affiliations:** 1 School of Food and Health, Beijing Advanced Innovation Center for Food Nutrition and Human Health, Beijing Engineering and Technology Research Center of Food Additives, Beijing Technology and Business University, Beijing 100048, China; 2 School of Food Science and Technology, State Key Laboratory of Food Science and Technology, Jiangnan University, Wuxi 214122, China; 3 Beijing Yushiyuan Food Co., Ltd., Beijing 101407, China

**Keywords:** white kidney bean, hepatic steatosis, gut microbiota, fecal metabolites, protective effects

## Abstract

Disturbances in the gut microbiota and its derived metabolites are closely related to the occurrence and development of hepatic steatosis. The white kidney bean (WKB), as an excellent source of protein, dietary fiber, and phytochemicals, has recently received widespread attention and might exhibit beneficial effects on a high-fat diet (HFD)-induced hepatic steatosis via targeting gut microbiota and its metabolites. The results indicated that HFD, when supplemented with WKB for 12 weeks, could potently reduce obesity symptoms, serum lipid profiles, and glucose, as well as improve the insulin resistance and liver function markers in mice, thereby alleviating hepatic steatosis. An integrated fecal microbiome and metabolomics analysis further demonstrated that WKB was able to normalize HFD-induced gut dysbiosis in mice, thereby mediating the alterations of a wide range of metabolites. Particularly, WKB remarkably increased the relative abundance of probiotics (*Akkermansiaceae*, *Bifidobacteriaceae*, and *norank_f_Muribaculaceae*) and inhibited the growth of hazardous bacteria (*Mucispirillum*, *Enterorhabdus*, and *Dubosiella*) in diet-induced hepatic steatosis mice. Moreover, the significant differential metabolites altered by WKB were annotated in lipid metabolism, which could ameliorate hepatic steatosis via regulating glycerophospholipid metabolism. This study elucidated the role of WKB from the perspective of microbiome and metabolomics in preventing nonalcoholic fatty liver disease, which provides new insights for its application in functional foods.

## 1. Introduction

Non-alcoholic fatty liver disease (NAFLD) has been drastically threatening public health worldwide since it has a global incidence of more than 25% with an increasing rate of prevalence [1]. As a chronic disease, NAFLD is mainly characterized by excessive lipid accumulation in hepatocytes and encompasses a proceeded series of liver histologic changes on a continuum from a milder condition, such as dyslipidemia leading to hepatic lipid accumulation, and subsequently to the relatively severe hepatic steatosis with hepatocyte damage and inflammation [2]. Currently, the occurrence and development of NAFLD have been reported to be affected by multiple factors, such as genetic, metabolic, and microbiome-related factors [3]. Due to its complex etiopathogenesis, no effective drugs are approved for the treatment of NAFLD worldwide. Thus, it is urgent to explore new therapeutic strategies to reduce the risk of NAFLD.

Numerous studies have revealed that gut microbiota and associated disorders are important regulators in the pathophysiology of NAFLD since the liver and intestine are connected anatomically through the hepatic portal system [4,5]. Furthermore, it was found that both the Western-style diet and high fructose load did not trigger the liver steatosis of germ-free (GF) mice, suggesting that diet-induced experimental liver steatosis depends in multiple ways on gut microbiota [6]. Notably, gut microbiota from a high-fat diet (HFD)-responder donor predisposed transplanted GF mice to NAFLD [7,8]. These results provided scientific support for targeting the gut microbiota as a protective strategy against the development of diet-related NAFLD. In addition, it was found that microbiota-derived metabolites were capable of crossing the epithelial barrier to enter the circulation and modulate NAFLD progression [9]. Intriguingly, there is increasing evidence that gut microbes can use functional foods or ingredients as precursors to produce large amounts of beneficial metabolites, which, in turn, can effectively intervene in NAFLD [10,11]. Therefore, gut microbial metabolites are deemed important mediators in maintaining the balance of the gut–liver axis, highlighting the involvement of host-diet–microbiota–metabolite interactions in NAFLD.

To date, many health organizations around the world have called for serious changes in dietary patterns to improve the possible health problems caused by NAFLD, especially by promoting the consumption of plant-based diets [12]. Among dietary approaches, legumes have been increasingly explored as a nutritional, safe, and feasible whole-food-based strategy for NAFLD treatment owing to their low energy density and high nutrient density [13,14,15]. White kidney beans (*Phaseolus vulgaris* L.), the most consumed legume in the world, have attracted much attention recently due to their functional ingredients [16]. Specifically, its protein, polysaccharide, and other bio-components have been demonstrated to have various health benefits, such as hypoglycemic [17,18] and hypolipidemic effects [19,20] and repairing intestinal function [21]. Based on the available published reports, previous studies on the biological activity of white kidney beans have mainly focused on their single components or extracts. Furthermore, most of the functional components in whole white kidney beans are indigestible and/or non-absorbable, thus evading the digestion of the upper gastrointestinal tract. In this context, they could reach the intestine intact to interact with the gut microbiota. To date, the mode of interaction between the whole white kidney bean and gut microbiota and its role in the prevention of diet-induced hepatic steatosis have not been published. Noticeably, multiple components of whole foods have been shown to have potentially synergistic effects [22,23], and there is a growing trend to consume whole white kidney beans. Therefore, these findings have prompted us to explore the improvement effects of whole white kidney beans on diet-induced NAFLD from the perspective of gut microbiota-metabolites strategies.

Herein, we hypothesized that the whole white kidney bean could effectively alleviate diet-induced NAFLD by targeting the regulation of gut microbiota and its metabolites. To clarify this hypothesis, in this study, we aimed to (1) examine the effects of the whole white kidney bean on the alleviation of NAFLD by determining levels in the physiological, histological, and blood biochemistry parameters; (2) explore the gut microbiota modulatory effects of the whole white kidney bean by the high-throughput pyrosequencing of 16S rRNA; (3) analyze the changes in specific feces metabolites after whole white kidney bean supplementation based on untargeted metabolomics.

## 2. Materials and Methods

### 2.1. Materials

White kidney (*Phaseolus vulgaris* L.) beans (WKB) were provided by Beijing Yushiyuan Food Co., Ltd. (Beijing, China) and prepared as follows. In detail, the washed beans were steamed in a steam chamber for 40 min after soaking in distilled water for 4 h at room temperature. Then, the cooked beans were freeze-dried, ground into powders, sieved through an 80-mesh screen, and stored at −20 °C until use. The diets for animal experiments were obtained from SYSE Biotech. Co., Ltd. (Changzhou, China).

### 2.2. Animal Experimental Design and Diets

Forty male C57/BL 6J mice (specific-pathogen-free (SPF) grade, 18∼20 g, 6 weeks) were purchased from the Vital River Laboratory Animal Technology Co., Ltd. (Beijing, China). The mice were housed in the SPF condition at a room temperature of 23 ± 2 °C under controlled humidity (50 ± 5%) with a 12 h light/dark cycle. The mice were raised in four per cage with free access to water and diets.

After acclimatization for one week, the mice were randomly divided into four groups (*n* = 8/group) and received one of the following diets: (1) A normal control diet (NCD group); (2) A normal control diet supplemented with 20% WKB power group (NCD-WKB group); (3) A high-fat diet group (HFD); (4) A high-fat diet supplemented with 20% of the WKB power group (HFD-WKB). Noticeably, mice in the NCD and NCD-WKB groups (10% energy derived from fat, 3.85 kcal g^−1^), as well as in the HFD and HFD-WKB groups (60% energy derived from fat, 5.24 kcal g^−1^), were fed diets with different energy densities. The 20% cooked WKB was selected as an effective dosage for protection against HFD-induced hepatic steatosis based on an achievable and physiologically relevant level of the legume intake in humans as suggested in the Dietary Guidelines for Chinese Residents (2022) and the results of previous studies [24]. Furthermore, to ensure that each macronutrient contributed equally to the caloric density of these diets, the nutrients of experimental diets were adjusted according to the proximate nutritional composition of WKB. In this context, NCD and NCD-WKB, HFD and HFD-WKB diets were isocaloric, respectively. The detailed nutrient compositions of WKB and experimental diets are shown in Table S1.

Body weight and food intake were recorded weekly during the experimental period. After 12 weeks of dietary intervention, all mice were fasted overnight. Then, they were anesthetized (pentobarbital) for blood collection from the orbit and sacrificed by cervical dislocation. Serum samples were obtained after the centrifugation of blood samples at 3000 rpm/min for 10 min, which were stored at −80 °C. The white adipose tissue and livers were harvested and weighed. In addition, partial liver samples were fixed in 4% paraformaldehyde for histological analysis, and the remaining samples were immediately frozen in liquid nitrogen and stored at −80 °C.

The animal experimental protocol was approved by the Institutional Animal Care and Use Committee of China Agricultural University and complied with the experimental animal welfare standard (Protocol number: AW90303202-5-1).

### 2.3. Oral Glucose Tolerance Test (OGTT)

An OGTT was usually performed three days before the end of the experiment, as previously described [25]. In detail, all mice were fasted for 6 h at 12 weeks. According to the body weight, each mouse received a glucose solution (2 g/kg body weight) by oral gavage after the determination of fasted blood glucose levels. Subsequently, the collected glucose levels of blood samples from the tail vein at 30, 60, 120, and 150 min were immediately measured using a glucometer (Bayer Contour, Tarrytown, NY, USA). The glucose tolerance of each group was evaluated by the calculated area under the curve (AUC) for blood glucose.

### 2.4. Biochemical Assays

The levels of serum total cholesterol (TC), triglyceride (TG), high-density lipoprotein cholesterol (HDL-C), low-density lipoprotein cholesterol (LDL-C), fasting blood glucose (FBG), alanine aminotransferase (ALT), and aspartate aminotransferase (AST) were measured by a 3100-automatic biochemistry analyzer (Hitachi Ltd., Tokyo, Japan). In addition, the concentrations of TG and TC in liver tissues were detected using commercial kits (Nanjing Jiancheng Bioengineering Institute, Nanjing, China). All tests were completed according to the manufacturer’s instructions.

### 2.5. Histological Analysis

The histomorphometric analysis of liver tissues was conducted based on a previous method [26]. Briefly, the fixed liver tissues in 4% paraformaldehyde were dehydrated and embedded in wax and then sectioned in 5 μm slices. These sections were routinely stained with hematoxylin and eosin (H&E). In addition, the Oil Red O staining was performed to evaluate lipid accumulation in the liver. Then, the liver tissue samples were embedded with an optimum cutting temperature (OCT) compound and sliced. The obtained 8-μm-thick frozen sections were subjected to Oil Red O staining. These slices were examined, observed, and imaged using a NIKON Eclipse CI microscope (Nikon, Tokyo, Japan).

### 2.6. Gut Microbiota Sequencing and Analysis

The fresh feces were collected from each mouse in different groups at week 12 for 16 srRNA gene sequencing. The total genomic DNA was extracted from the fecal samples using the OMEGA Soil DNA kit (Omega Bio-Tek, Norcross, GA, USA) and following the protocol of the manufacturer. The NanoDrop ND-1000 spectrophotometer (Thermo Fisher Scientific, Wilmington, DE, USA) and agarose gel electrophoresis were used to monitor the quantity and quality of extracted genomic DNA samples, respectively. The V3–V4 hypervariable region of 16S rRNA genes was selected for generating amplicons by the polymerase chain reaction (PCR), using the forward primer 338F (5′-ACTCCTACGGGAGGCAGCAG-3′) and the reverse primer 806R (5′-GGACTACHVGGGTWTCTAAT-3′) [27]. Subsequently, the mixed PCR products were extracted from a 2% agarose gel and purified with an AxyPrep DNA Gel Extraction Kit (Axygen Biosciences, Union City, CA, USA). After being quantified individually, the obtained amplicons were pooled in equal amounts, and pair-end 2 × 300 bps sequencing was performed on an Illumina MiSeq platform in accordance with the standard protocols at Shanghai Majorbio Bio-Pharm Technology Co., Ltd. (Shanghai, China).

After sequencing was complete, the raw reads underwent a series of processes, including quality filtering, trimming, de-noising, merging to eliminate the adapter pollution, and removing low-quality reads. The remaining high-quality reads were then clustered into the same operational taxonomic units (OTU) with a 97% similarity cutoff using Uparse (Version 7.0, http://www.drive5.com/uparse/, accessed on 22 April 2022). According to the clustering and annotation results of OTU, information on the microbial species and their relative abundances could be obtained. Diversity metrics, including α- and β-diversity, were calculated using the core-diversity plugin by Mothur (V 1.30.2) and QIIME2 (version 1.7.0), which were visualized with R software (version 2.15.3). The Principal Coordinates Analysis (PCoA) was conducted to assess the β-diversity based on the unweighted UniFrac distances. An ANOSIM statistic test was used to determine the significant differences in β-diversity. In addition, an unweighted pair-group method with an arithmetic mean (UPGMA) was performed to calculate the hierarchical clustering of the samples. The biomarker taxa with a significantly varied relative abundance among different groups (*p* < 0.05) were identified using linear discriminant analysis (LDA) coupled with the effect size analysis (LEfSe) with a threshold of 3.0.

### 2.7. Untargeted Metabolomics Analysis

Ultra-high performance liquid chromatography-tandem mass spectrometry (UPLC-MS/MS) was used to analyze the metabolites in fecal samples. Briefly, 50 mg of feces were added to 0.4 mL of a pre-cooled methanol and acetonitrile mixture solution (1:1, *v*/*v*), which was vortexed, homogenized, and sonicated; the 0.2 mg/mL of L-2-chlorophenylalanine solution as an internal standard was added and the supernatant was taken by centrifugation. The precipitated proteins were removed after the supernatant samples were incubated at −20 °C for 30 min. Subsequently, the supernatant residue after the nitrogen evaporation treatment was re-dissolved by adding 100 μL of the pre-chilled ethanol and acetonitrile mixture. Finally, the sample supernatant, after filtration through the 0.22 μm membrane, was used for further UPLC-MS/MS analysis. The analysis parameters were as described previously [28].

Pogenesis QI 2.3 (Nonlinear Dynamics, Waters, Milford, MA, USA) were used to preprocess the acquired raw data of mass spectroscopy, including data filtering, peak identification, missing value estimation, folding change, and data normalization. The annotation and identification of fecal metabolites were carried out through the Kyoto Encyclopedia of Genes and Genomes (KEGG, https://www.genome.jp/kegg/pathway.html, accessed on 5 September 2022) and Human Metabolome Database (HMDB; https://hmdb.ca/metabolites, accessed on 5 September 2022). Principal component analysis (PCA) and orthogonal partial least squares discriminant analysis (OPLS-DA) were conducted using the R software package MetaboAnalystR. The variable importance of the projection (VIP) > 1.0, *p*-value < 0.05 (two-tailed student’s *t*-test), and fold change (FC) ≥ 1.5 or FC ≤ 0.5 were considered to indicate differentially expressed metabolites among the different groups. According to the identified and differential biomarkers, the annotation and enrichment analysis were conducted on the main signal transduction pathways.

### 2.8. Statistical Analysis

The experimental data were statistically analyzed and graphed using Graphpad Prism 8.0 (GraphPad Software, San Diego, CA, USA). An unpaired student’s *t*-test was used for comparisons between the two groups, while a one-way analysis of variance (ANOVA) following Tukey’s multiple comparisons test was conducted to assess the differences among three groups or more. Data were expressed as the mean ± standard error of the mean (SEM). A *p*-value < 0.05 was deemed to be statistically significant.

## 3. Results

### 3.1. WKB Improved the Obese Phenotypes and Liver Function in HFD-Fed Mice

**Figure 1:**
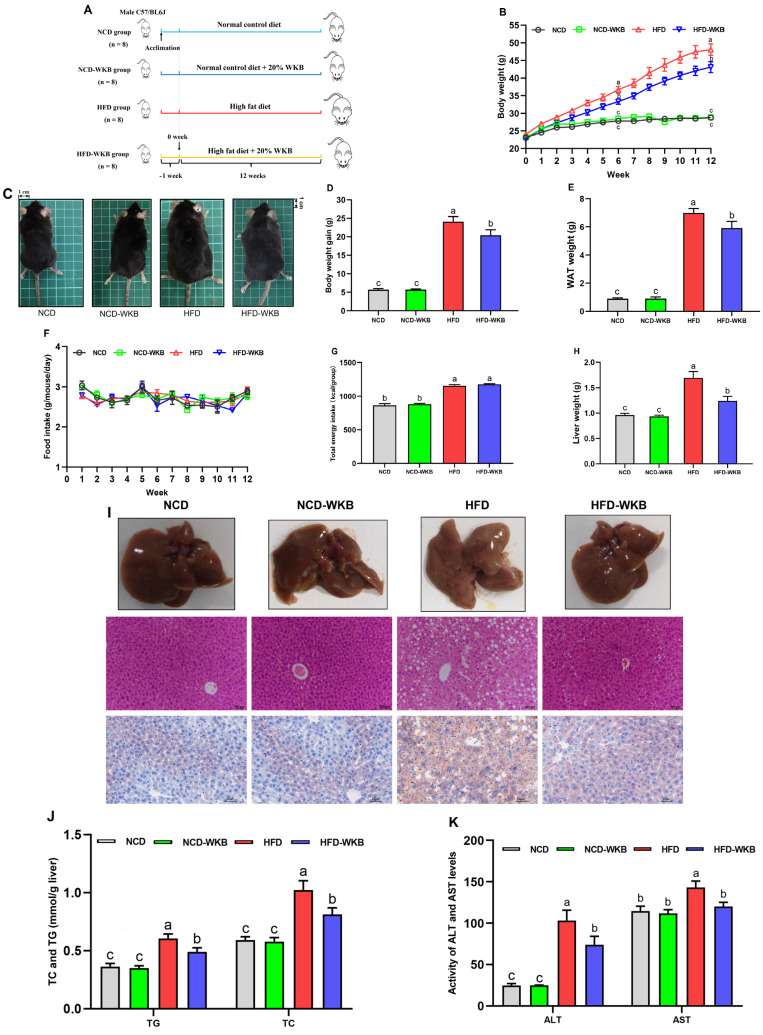
WKB improved the obese phenotypes and liver function in HFD−fed mice. (**A**) Illustration of animal experimental design. (**B**) Body weight changes. (**C**) Representative appearance of mice. (**D**) Body weight gain. (**E**) White adipose tissues (WAT) weight. (**F**) Food intake changes. (**G**) Total energy intake. (**H**) Liver weight. (**I**) Morphology (**top**, 200 × magnification), H&E, and Oil Red O staining (**bottom,** 400 × magnification) of the representative livers. (**J**) TG and TC contents in the livers. (**K**) ALT and AST levels in the serum. The different letters on the top of graph bars represented statistical significance (*p* < 0.05).

The mice were treated with WKB for 12 weeks to explore its improvement effects on the obese phenotypes and hepatic steatosis in HFD-fed mice (Figure 1A). The results showed that from week 6, WKB significantly reduced the body weight in HFD-fed mice (Figure 1B), as evidenced by the respective appearance of mice at the end of the 12 weeks (Figure 1C). Furthermore, the body weight gains and WAT weight of the HFD-WKB group were significantly lower than those of the HFD group (Figure 1D,E). Notably, no significant differences were observed in the food intake and total energy intake between the HFD and HFD-WKB groups (Figure 1F,G).

As can be derived from Figure 1H, 12 weeks of HFD significantly increased the liver weight of the mice compared to the NCD. As expected, WKB significantly prevented an increase in liver weight, suggesting that it might have a good improvement on NAFLD. To further verify this claim, the representative liver samples were collected and photographed, as shown in Figure 1I. It was found that the NCD and NCD-WKB group showed dark red livers, while the HFD group showed light yellow livers and lost their blood color, which was considered to be a typical visual manifestation of hepatic steatosis. After 12 weeks of WKB supplementation, the dark brownish-red color of the livers was significantly restored. Moreover, the results of H&E and Oil Red O staining showed that HFD resulted in obvious liver injuries, including lipid deposition, different degrees of steatosis with the infiltration of inflammatory cells in hepatocytes, and an irregular hepatocytes arrangement with a balloon-like structure, which were markedly weakened by WKB. These results were consistent with significantly decreased TC and TG contents in the livers (Figure 1J). Meanwhile, the significantly lowered serum ALT and AST levels further supported these findings (Figure 1K). Noticeably, for the above indices, no significant differences were observed between the NCD and NCD-WKB groups.

### 3.2. WKB Alleviated Dyslipidemia and Hyperglycaemia in HFD-Fed Mice

**Figure 2:**
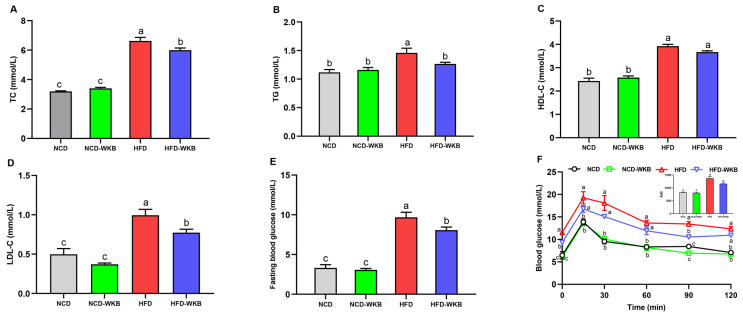
Effects of WKB on the serum lipid profiles, fasting blood glucose, and OGTT in HFD-fed mice. (**A**) TC. (**B**) TG. (**C**) HDL-C. (**D**) LDL-C. (**E**) Fasting blood glucose. (**F**) OGTT. The means marked with different superscript letters represent statistical significance (*p* < 0.05).

The development of NAFLD is strongly associated with abnormal blood lipids, blood glucose, and glucose tolerance. Mice in the HFD group presented significantly higher serum lipid levels than those of mice in the NCD group. WKB supplementation significantly lowered the HFD-mediated upregulation of serum TC, TG, and LDL-C (Figure 2A,B,D), whereas there was no significant difference in the serum HDL-C level between HFD and HFD-WKB groups. In addition, the elevated fasting blood glucose level caused by HFD fed was remarkably decreased by WKB (Figure 2E). The improvement of OGTT (Figure 2F) further demonstrated that WKB could effectively decrease the insulin resistance of HFD-fed mice, thus contributing to the alleviation of NAFLD.

### 3.3. WKB Altered the Diversity of Gut Microbiota in Mice with HFD-Induced NAFLD

**Figure 3:**
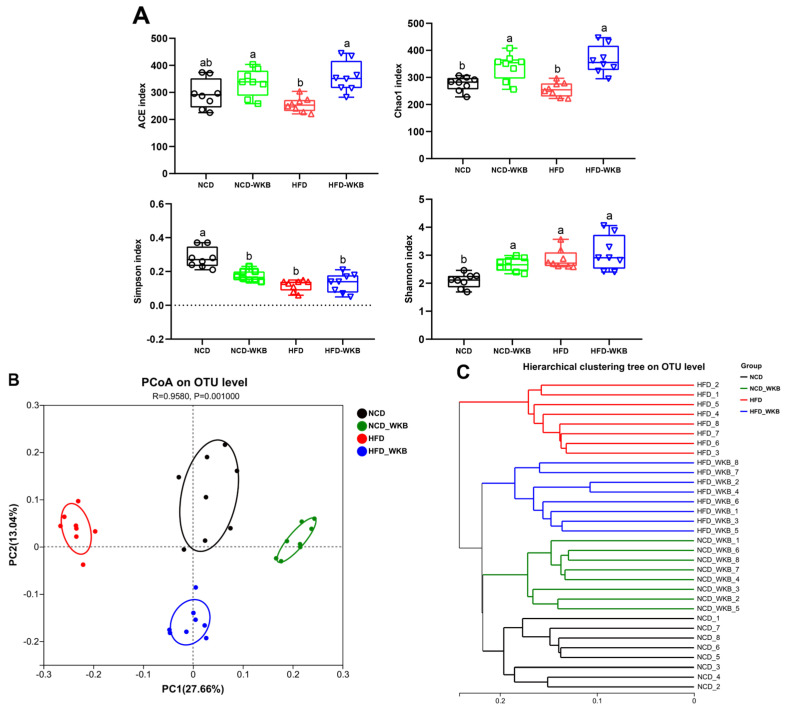
WKB altered the overall structure of the gut microbiota. (**A**) α−Diversity. (**B**) PCoA. (**C**) Hierarchical clustering. The different letters on the top of the graph bars represent statistical significance (*p* < 0.05).

The results of 16S rRNA sequencing analysis showed that a total of 1,594,675 high-quality sequences were obtained from 32 samples with an average of 49,833 sequences. Both the Rarefaction curve and Shannon index curve demonstrated that the sequencing depth was sufficient and the vast majority of diversity and rare new phylotypes could be covered (Figure S1A,B). The α-diversity of microbial communities, including richness (ACE and Chao1 indexes) and diversity (Shannon and Simpson indexes), is shown in Figure 3A. HFD feeding significantly lowered the microbial community richness of mice compared with that of the NCD group but had no significant effects on the microbial community diversity of mice. These negative effects were markedly alleviated after 12 weeks of WKB supplementation. In addition, β-diversity was used to evaluate the similarity of microbial communities among the different groups. As illustrated in Figure 3B, PCoA showed that the distribution of the microbial community in the NCD-WKB and HFD-WKB groups was clearly separated from NCD and HFD groups, respectively, as evidenced by the ANOSIM test (R = 0.9580, *p* = 0.001). Similarly, hierarchical clustering analysis demonstrated that the different groups aggregated into four branches (Figure 3C). According to the above results, it was found that WKB could significantly alter the microbiota structures of the NCD and HFD groups, which was beneficial when alleviating the gut microbiota dysbiosis induced by HFD.

### 3.4. WKB Modulated the Composition of Gut Microbiota in Mice with HFD-Induced NAFLD

**Figure 4:**
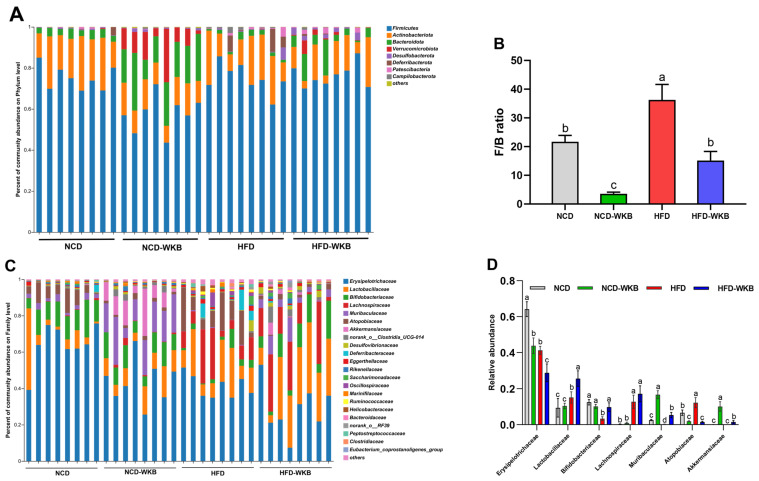

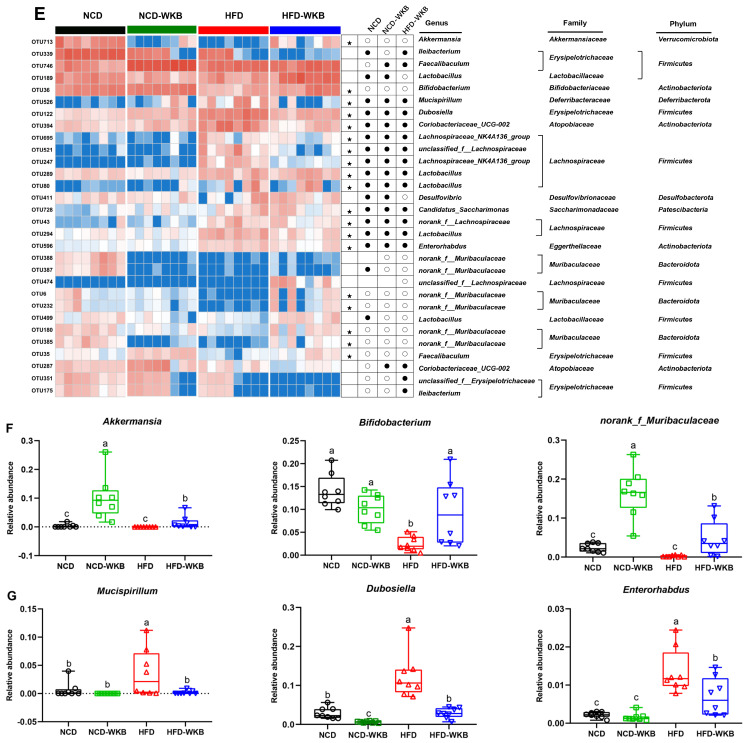
Effects of WKB on gut microbial community. (**A**) Phylum−level. (**B**) *Firmicutes*/*Bacteroidetes* (F/B) ratio. (**C**) Family−level. (**D**) Relative abundance of key gut microbiota at the family level. (**E**) Heatmap of the most changed 30 OTUs. (**F**,**G**) Relative abundance of key genus. The relative abundances of OTUs in the NCD or HFD−WKB groups were higher and lower than those in the HFD group, represented by white circles (○) and black dots (●), respectively. When the relative abundance of OTUs in the NCD group was changed by HFD and then reversed by WKB, it was marked with a black star (★). The different letters on the top of graph bars represented statistical significance (*p* < 0.05).

To further elucidate the effects of WKB on the gut microbiota composition, the main differences at the phylum, family, and genus levels were analyzed. At the phylum level, *Firmicutes*, *Bacteroidota*, *Actinobacteriota*, and *Verrucomicrobiota* were found to be the most dominant phyla in the four groups (Figure 4A). Although no significances were observed in these bacterial phyla between the NCD and HFD groups, WKB significantly increased the relative abundance of *Bacteroidota* and *Verrucomicrobiota* compared with those of the HFD group (Figure S2). Furthermore, the remarkable up-regulated *Firmicutes*/*Bacteroidetes* (F/B) ratio in HFD-fed mice was significantly reduced by WKB (Figure 4B). Consistent results were also found between the NCD and NCD-WKB groups. At the family level, the relative abundance of *Lactobacillaceae*, *Bifidobacteriaceae*, *Muribaculaceae*, and *Akkermansiaceae* in the HFD-WKB group was significantly higher than those of the HFD group, while the relative abundance of *Erysipelotrichaceae* and *Atopobiaceae* was markedly reduced after WKB intervention (Figure 4C,D).

In addition, the most changed 30 OTUs were selected to excavate the key bacterial genera that responded to WKB supplementation. As shown in Figure 4E, 17 OTUs, and 11 OTUs were significantly increased and decreased by HFD in these altered 30 OTUs, respectively, when compared with the NCD group. However, the changed 19 OTUs (highlighted with black stars) were prevented after 12 weeks of WKB supplementation, including seven increased and twelve decreased OTUs. In detail, the comparative analysis results between the HFD-WKB and HFD groups showed that the relative abundance of *Akkermansia*, *Bifidobacterium*, and *norank_f_Muribaculaceae* was significantly promoted (Figure 4F) while the relative abundance of *Mucispirillum*, *Enterorhabdus*, and *Dubosiella* was remarkably reduced (Figure 4G). Specifically, the bacteria genera *Akkermansia*, *Bifidobacterium*, and *norank_f_Muribaculaceae* were subordinate to the family *Akkermansiaceae*, *Bifidobacteriaceae*, and *Muribaculaceae*, respectively, which were demonstrated to be significantly elevated by the WKB intervention (Figure 4C,D). Moreover, discriminant analysis (LDA) scores derived from LEfSe analysis showed consistent results, indicating that the key phylotypes of the gut microbiota in response to WKB supplementation were the bacterial genera *Akkermansia*, *Bifidobacterium*, and *norank_f_Muribaculaceae* (Figure S3). These results suggest that WKB could promote the boom of some beneficial bacteria and inhibit some HFD-dependent taxa to improve hepatic steatosis.

### 3.5. WKB Regulated Fecal Metabolic Profiles in HFD-Fed Mice

**Figure 5:**
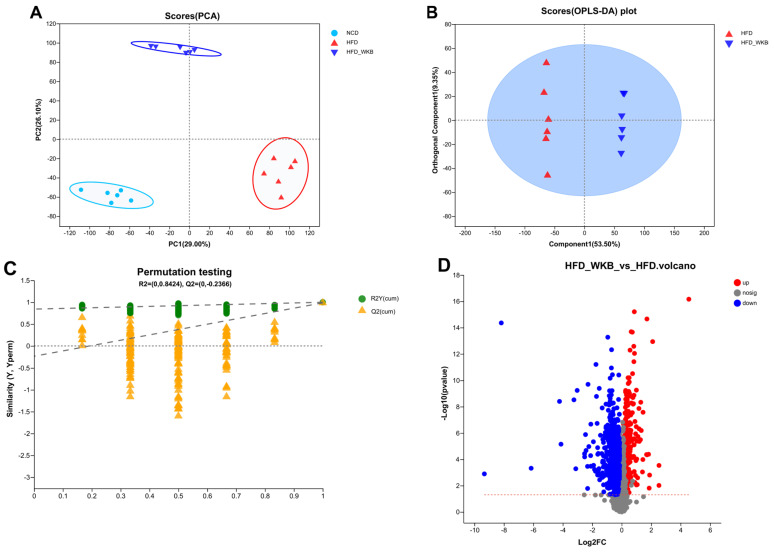

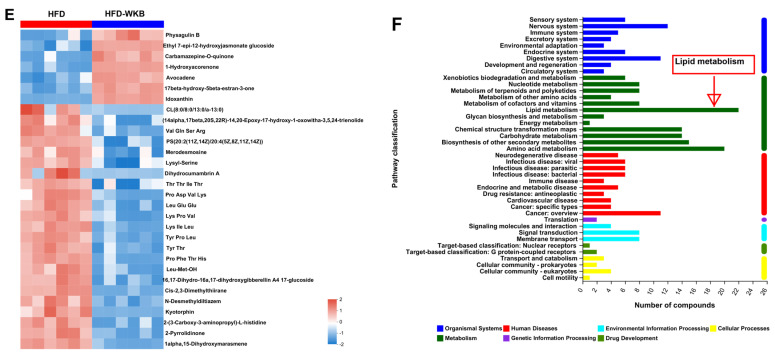
Effects of WKB on fecal metabolite profiles. (**A**) PCA score plots; (**B**) Scores plots of OPLS−DA between the HFD and HFD−WKB groups; (**C**) Permutation tests conducted with 200 random permutations in the OPLS−DA model. (**D**) Volcano plot of fecal metabolomics of mice showing the significantly changed metabolites in the HFD−WKB group compared to the HFD group. (**E**) Heatmap of 30 significantly altered metabolite elevels between the HFD and HFD−WKB groups. Blocks in red and blue denote high and low z-score values, respectively. (**F**) KEGG annotation analysis of the changed metabolites based on the differences between the HFD and HFD−WKB groups.

Microbial metabolites are believed to be an intermediate phenotype that mediates interactions between the host and microbiome, thus providing a reliable and effective way for gut microbiota activity. In this context, it was hypothesized that the potential mechanism of WKB on ameliorating NAFLD was related to the fecal metabolites modulated by the gut microbiota. To evaluate the metabolic alternations in response to the gut microbiota and remodeled by WKB supplementation, an untargeted metabolomics analysis was performed to identify the changed fecal metabolites. The PCA data distribution revealed that the structure of these identified metabolite profiles was separated from each other among different groups (Figure 5A). Moreover, for the HFD and HFD-WKB groups, scores scattered plots of OPLS-DA between them, which exhibited a distinct separation in the mixed ion modes (Figure 5B). The further permutation test indicated that the models were very stable and highly predictable when explaining the metabolite variations between the HFD-WKB and HFD groups.

After the peak alignment and removal of unidentified peaks, a total of 1040 metabolites with definite information (VIP values > 1.0, fold change > 1.0 or <1.0, and *p* values < 0.05) were identified between the HFD and HFD-WKB groups in both positive and negative ion modes. As shown in Figure 5C, compared with the HFD group, 318 and 722 altered metabolites in the HFD-WKB group were up-regulated and down-regulated, respectively (Figure 5D). Subsequently, the top 30 significantly altered metabolites caused by WKB were selected for heat map analysis (Figure 5E). WKB could effectively prevent the altered metabolic features caused by HFD, mainly including lipids and lipid-like molecules, organoheterocyclic compounds, organic acids, derivatives, and benzene. Moreover, to achieve a deeper understanding of the metabolic alterations in response to WKB supplementation, the metabolic pathway enrichment analysis was conducted using MetaboAnalyst based on the KEGG database. The results showed that those differential metabolites between the HFD-WKB and HFD groups were annotated into seven categories of KEGG metabolic pathways with lipid metabolism being the most significantly enriched (Figure 5F).

**Figure 6:**
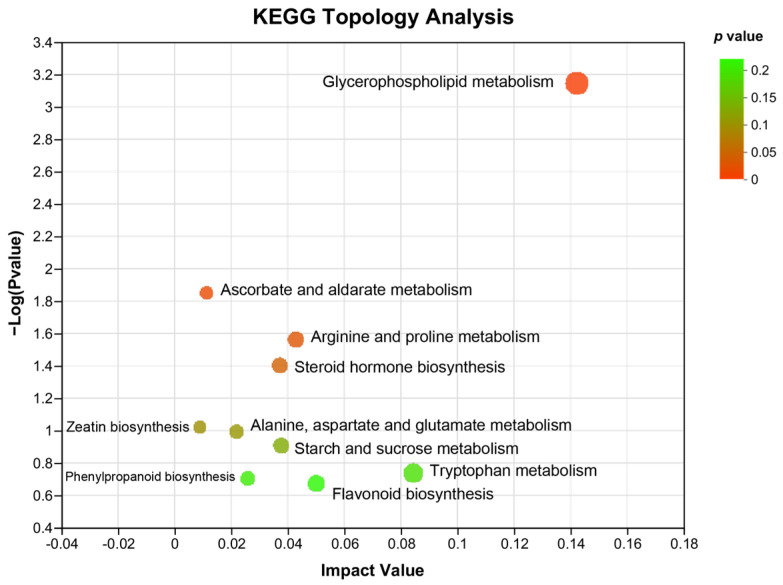
KEGG topology analysis. The path impact value and *p*−value are reflected by the size and color of each circle, respectively.

In addition, KEGG topology analysis revealed that compared with that of HFD-induced NAFLD mice, the major metabolic pathways that were changed by WKB included glycerophospholipid metabolism, ascorbate, and aldarate metabolism, arginine and proline metabolism, steroid hormone biosynthesis, zeatin biosynthesis, alanine, aspartate and glutamate metabolism, starch, and sucrose metabolism, phenylpropanoid biosynthesis, flavonoid biosynthesis, and tryptophan metabolism. Among these, the glycerophospholipid metabolism and ascorbate and aldarate metabolism were significantly altered metabolic pathways (Figure 6), which could be closely related to the alleviation effects of WKB on diet-induced hepatic steatosis.

## 4. Discussion

The high prevalence of NAFLD has posed a serious threat to human health. The excessive consumption of high-fat foods is considered a key factor in the formation and development of NAFLD. Emerging evidence has suggested that legumes, as excellent sources of protein, dietary fiber, and polyphenols, possess natural protective effects against NAFLD through multiple pathways [29,30]. As expected, WKB demonstrated a significant hepatoprotective effect against HFD-induced NAFLD, as evidenced by reductions in body weight and lipid profile levels, as well as the normalization of liver function markers and insulin resistance. A previous study showed that protein hydrolysates obtained from WKB had an in vitro cholesterol-lowering activity [19]. In line with this result, its daily supplementation with protein hydrolysate from WKB could effectively alleviate the impairment of hepatic injury in BALB/c mice fed with a high-fat, high-cholesterol diet [31]. Noticeably, in this study, the lower alanine aminotransferase and low-density lipoprotein cholesterol levels were found in animals fed with whole bean flour compared to that of bean protein hydrolysate. Furthermore, a growing body of evidence has demonstrated that WKB extracts (α-Amylase inhibitors) could significantly improve hepatic steatosis in HFD-fed mice [32,33]. In addition to protein and WKB extracts, dietary fiber might play an important role in the protective effects of WKB against diet-induced hepatic steatosis. In a previous study, the dietary fiber of kidney beans was proven to be responsible for its beneficial effects on reducing the total serum cholesterol in rats [34]. Thus, the synergies between these nutrients might be an important explanation for WKB in alleviating diet-induced hepatic steatosis in mice. However, these effects were not due to the differences in feed and energy intake. Thus, the protection mechanism of WKB on NAFLD remains elusive.

Numerous studies have illustrated the crucial role of gut microbiota and its metabolites in the pathological process of NAFLD [35,36]. Therefore, the regulation of gut microbiota and its associated fecal metabolites via functional foods or ingredients could mitigate NAFLD. The high content of dietary fibers, protein, and polyphenols in WKB enables it to regulate the gut microbiota and its related metabolites. In this study, some specific gut bacteria species and fecal metabolites were identified through the integrative analysis of the microbiome and metabolome after 12 weeks of WKB intervention, which could have contributed to the improvement in NAFLD. It has been shown that NAFLD is closely associated with the increased relative abundance of *Firmicutes* and decreased relative abundance of *Bacteroidota* and *Verrucomicrobiota*, as well as the elevated F/B ratio [37], which was further confirmed in this study. In particular, most studies have supported the claim that *Bacteroidetes* species possess various genes for carbohydrate-degrading enzymes [38], which could be beneficial in preventing the development of NAFLD. Our results suggest that WKB supplementation significantly promoted the bloom of *Bacteroidota* and *Verrucomicrobiota* and decreased the F/B ratio. Similarly, at the family level, the relative abundance of *Lachnospiraceae*, *Bifidobacteriaceae*, *Muribaculaceae*, and *Akkermansiaceae* was negatively correlated with the onset of diet-induced hepatic steatosis [39,40], which was in agreement with the results of this study. Conversely, WKB supplementation significantly elevated their relative abundance. In addition, as well-known probiotics, *Akkermania* and *Bifidobacterium* are one of the key genera of *Akkermansiaceae* and *Bifidobacteriaceae*, respectively. More importantly, *Akkermania* and *Bifidobacterium* showed the most consistent association with protection against the onset and development of NAFLD at the genus level [41]. Meanwhile, it was reported that the antihyperlipidemic effect of flavonoids from whole-grain oat was strongly correlated with the two bacteria and the genus *norank_f_Muribaculaceae* [42]. In the present study, the increased proportion of *Akkermansiaceae*, *Bifidobacteriaceae*, and *norank_f_Muribaculaceae* was in line with the improvement of HFD-induced NAFLD phenotypes in mice. Currently, some components of WKB have been demonstrated to potentially stimulate the growth of probiotics in the gut. The results of a 4-month randomized double-blinded placebo-controlled trial showed that, after the consumption of a 1.5 mg white kidney bean extract (WKBE, known as a-amylase inhibitor) for 4 months, participants possessed a significantly higher abundance of *Bifidobacterium* [43]. In animal studies, WKBE was found to attenuate HFD-induced obesity and hepatic steatosis, which was deemed to be associated with the increased relative abundance of *Bifidobacterium* and *Akkermansia* [32,33]. However, a recent study showed discrepant results. A randomized placebo-controlled crossover trial of 20 healthy adults showed that 1 week of WKBE supplementation elevated the relative abundance of *Bacteroidetes* and reduced that of *Firmicutes*; however, no significant differences were observed in the gut microbiota composition between the WKBE and control treatment groups [44]. The duration of this intervention might be the main reason for different results. As such, it is, thus, reasonable to believe that the regulation of these beneficial bacterial genera is partly responsible for the efficacy of WKB due to their significantly increased relative abundances. On the other hand, some HFD-dependent bacteria, such as *Mucispirillum*, *Enterorhabdus*, and *Dubosiella*, are deemed to be negatively correlated with the pathogenesis and progression of NAFLD [45,46]. WKB significantly decreased their relative abundance to some extent, which might help to alleviate NAFLD. Overall, the potent alleviation of WKB on NAFLD could be at least partially attributed to the increased relative abundance of beneficial bacteria and reduced hazardous bacteria.

In addition, non-targeted metabolomics based on UHPLC-MS/MS was used to identify differential metabolites and possibly elucidate the metabolic pathways of WKB on NAFLD. Based on the analysis results of metabolomics, the significantly different metabolites identified between the HFD and HFD-WKB groups were mainly lipids and lipid-like molecules, organoheterocyclic compounds, organic acids and derivatives, and benzene. As expected, according to the KEGG annotation analysis of altered metabolites, lipid metabolism was the most significant. Furthermore, glycerophospholipid metabolism was the major metabolic pathway that was changed by WKB. Glycerophospholipids are the main components of cell membranes, which can also act as the targets of protein signaling molecules to maintain cellular normal physiological activities [47]. Lipidomic analyses revealed that glycerophospholipids exhibited an obvious correlation with lipid parameters in relation to hepatic steatosis [48]. Previous studies have shown that multiple functional ingredients, including the tartary buckwheat extract [49], *Mori Fructus* polysaccharides [50], and some traditional Chinese medicine (Eight Zhes Decoction) [51], could ameliorate NAFLD by modulating glycerophospholipid metabolism. Although the other altered metabolic pathways, such as ascorbate and aldarate metabolism and arginine and proline metabolism [52], have also been proven to regulate NAFLD, glycerophospholipid metabolism contributed the most to the protective mechanism of WKB. These results indicate that WKB could alleviate hepatic steatosis by being metabolized into small molecules through the gut microbiota, thus activating multiple metabolic pathways.

## 5. Conclusions

In summary, the present study demonstrates that white kidney beans present protective effects against high-fat diet-induced hepatic steatosis, as evidenced by the decreased obesity phenotype and serum lipid profile levels, as well as the improved in the liver function markers and insulin resistance. The beneficial effects of white kidney beans on high-fat diet-induced hepatic steatosis may be partially attributed to its improvement effect on gut microbiota dysbiosis by promoting the bloom of some beneficial bacteria (*Akkermansiaceae*, *Bifidobacteriaceae*, and *norank_f_Muribaculaceae*) and reducing the relative abundance of certain hazardous bacteria (*Mucispirillum*, *Enterorhabdus*, and *Dubosiella*). Moreover, some specific differential metabolites that are altered by the white kidney bean could alleviate hepatic steatosis by multiple metabolic pathways, including glycerophospholipid metabolism, ascorbate and aldarate metabolism, and arginine and proline metabolism. Overall, the white kidney bean might be an economically feasible strategy for alleviating non-alcoholic fatty liver disease, and this study provides some insights into the development of legume-based foods in functional food areas.

## Data Availability

The data presented in this study are available in this article.

## Supplementary Materials

The following supporting information can be downloaded at: https://www.mdpi.com/article/10.3390/nu15133033/s1, Figure S1: The Rarefaction curve (A) and Shannon index curve (B); Figure S2: Relative abundance of gut microbiota at the Phylum level. The different letters on the top of graph bars represented statistical significance (*p* < 0.05); Figure S3: Key phylotypes of the gut microbiota in response to WKB supplementation; Table S1: Nutrients composition of experimental diets.
