# Supplementary material for: Protective Effects of White Kidney Bean (Phaseolus vulgaris L.) against Diet-Induced Hepatic Steatosis in Mice Are Linked to Modification of Gut Microbiota and Its Metabolites
Source: Nutrients. 2023 Jul 4;15(13):3033. doi: 10.3390/nu15133033 (PMC10347063; doi:10.3390/nu15133033)
Supplement: Supplementary file 1 [file nutrients-15-03033-s001.zip › nutrients-2471154-supplementary.pdf]

Table S1. Nutrients composition of experimental diets [1].

Ingredient (g/kg)	NCD	NCD-WKB	HFD	HFD-WKB
White kidney bean	0	200	0	200
Casein, 80 Mesh	189.56	142.62	258.45	211.51
L-Cystine	2.84	2.84	3.88	3.88
Corn Starch	479.79	385.73	0	0
Maltodextrin 10	118.48	108.58	161.53	67.47
Sucrose	65.21	73.74	88.91	88.91
Cellulose, BW200	47.39	0.00	64.61	11.81
Soybean Oil	23.7	21.9	32.31	26.11
Lard	18.96	18.96	316.6	316.6
Mineral Mix	9.48	9.48	12.92	12.92
DiCalcium Phosphate	12.32	12.32	16.8	16.8
Calcium Carbonate	5.21	5.21	7.11	7.11
Potassium Citrate, 1 H_2O	15.64	15.64	21.32	21.32
Vitamin Mix, V10001	9.48	0.95	12.92	12.92
Choline Bitartrate	1.9	1.90	2.58	2.58
FD&C Yellow Dye #5	0.04	0.04	0	0
FD&C Blue Dye #1	0.01	0.01	0.07	0.07
Total	1000	1000	1000	1000
% Energy and their source				
Protein	20	20	20	20
Carbohydrate	70	70	20	20
Fat	10	10	60	60
Total	100	100	100	100

[1] To equalize protein, fat, dietary fiber, and carbohydrate contents (g/kg diet) between the NCD and NCD-WKB diets, HFD and HFD-WKB diets, casein, soybean oil, cellulose, and maltodextrin were reduced in the NCD-WKB and HFD-WKB diets, respectively. Proximate nutritional composition of WKB (dry weight basis, g/100 g): protein 23.47; fat 3.10; total dietary fiber 26.40; carbohydrate 42.64; ash 4.39.

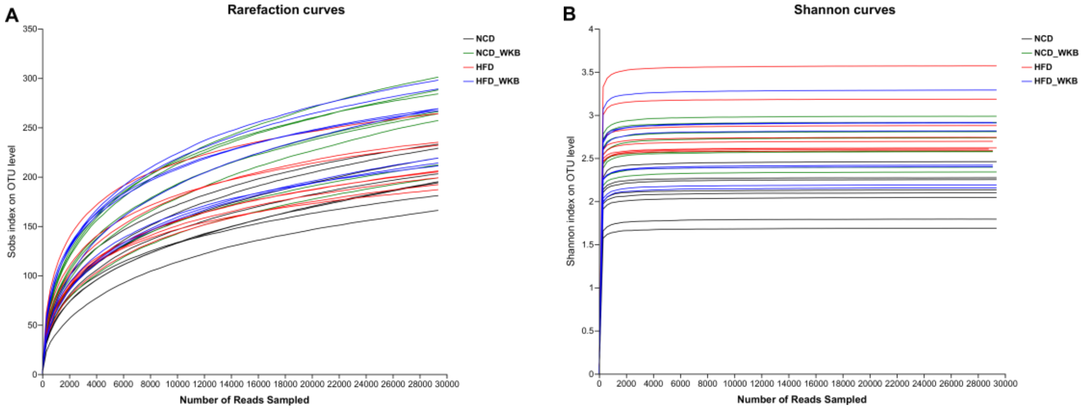

Figure S1. The Rarefaction curve (**A**) and Shannon index curve (**B**).

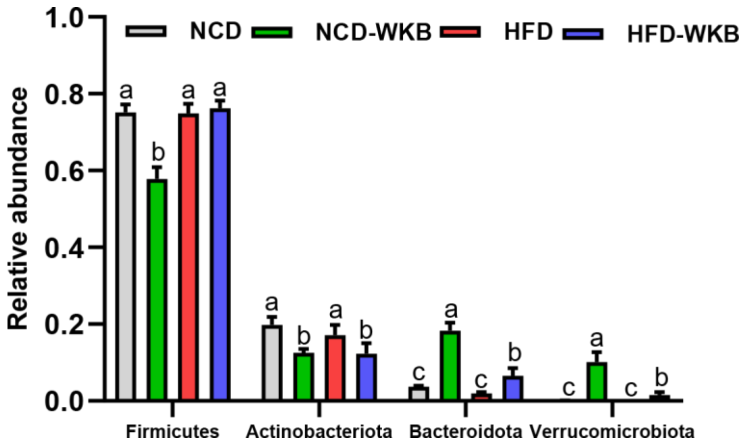

Figure S2. Relative abundance of gut microbiota at the Phylum level. The different letters on the top of graph bars represented statistical significance ($p < 0.05$).

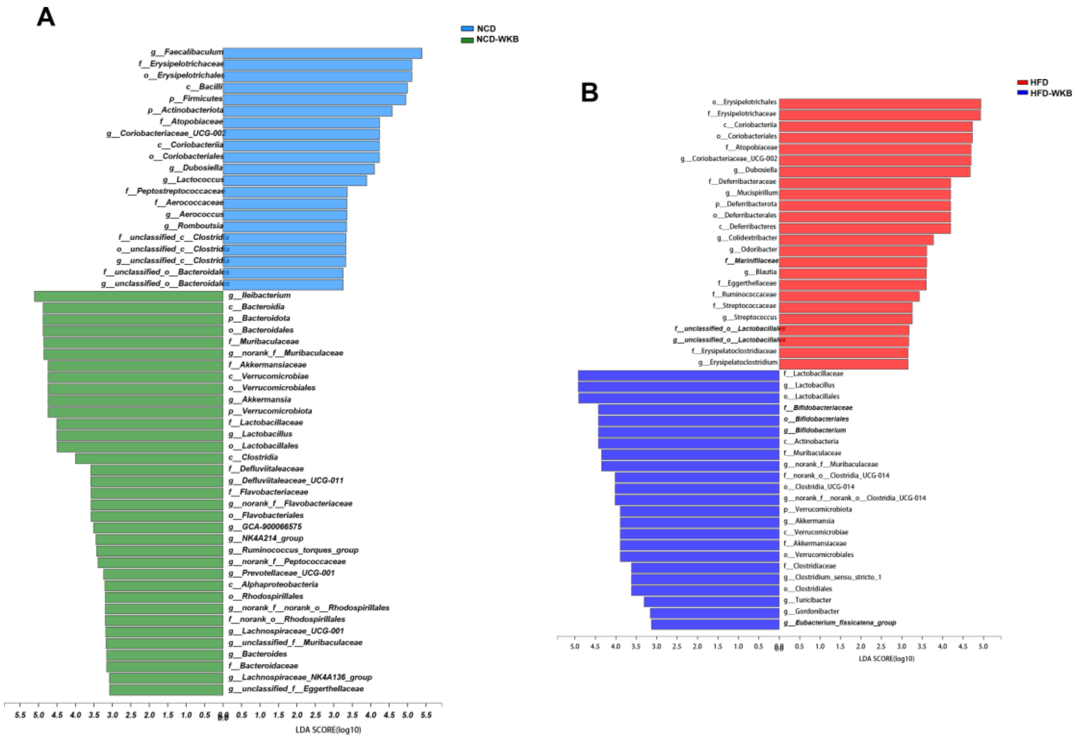

Figure S3. Key phylotypes of the gut microbiota in response to WKB supplementation.
